# Virology Downunder, a meeting commentary from the 2019 Lorne Infection and Immunity Conference, Australia

**DOI:** 10.1186/s12985-019-1217-6

**Published:** 2019-09-02

**Authors:** Gregor Ebert, Prasad N. Paradkar, Sarah L. Londrigan

**Affiliations:** 1 Infectious Disease and Immune Defence Division, The Walter and Eliza Hall Institute of Medical Research, Parkville, VIC Australia; 2 CSIRO Health and Biosecurity, Australian Animal Health Laboratory, Geelong, VIC 3220 Australia; 3 Department of Microbiology and Immunology, University of Melbourne, at the Peter Doherty Institute for Infection and Immunity, Melbourne, VIC 3000 Australia; 4 Department of Medical Biology, The University of Melbourne, Parkville, VIC 3010 Australia

**Keywords:** Infection, Immunity, Inflammation, Pathogenesis

## Abstract

The aim of this article is to summarise the virology content presented at the 9th Lorne Infection and Immunity Conference, Australia, in February 2019. The broad program included virology as a key theme, and the commentary herein highlights several key virology presentations at the meeting.

## Main text

The Lorne Infection and Immunity Conference is one of five scientific meetings held during each month of February in seaside town of Lorne, on the Great Ocean Road in Victoria (Australia). The specific aim of the meeting is to bring together basic, clinical and translational researchers - those who examine microbes and their impact on the innate or adaptive immune response, researchers who study the mechanisms that regulate immune responses, and those who apply this knowledge to preventing and treating infectious and inflammatory diseases. 2019 was the 9th Lorne Infection and Immunity Conference, convened by Heidi Drummer (Burnet Institute, Melbourne, Australia) and Paul Hertzog (Hudson Institute of Medical Research, Melbourne, Australia). The broad program included virology as a key theme, and the commentary herein highlights several key virology presentations at the meeting.

The ‘Infection and Inflammation’ session of the meeting was opened with a well-received presentation by Linfa Wang (Duke-NUS Medical School, Singapore) entitled ‘*Holy immune balance, batman*’, about the highly adapted immune response of bats to viral infections.

Bats have been characterized as an important reservoir of various zoonotic viruses including Nipah, SARS, MERS, Marburg and Ebola viruses [1], but remarkably are able to live asymptomatically with otherwise potentially lethal viruses [2]. Wang and colleagues are interested in understanding the underlying immune mediated regulatory mechanisms that facilitate a highly effective balance between viral defense and tolerance in bats as viral hosts.

During their evolutionary adaption to effective flight, bats not only developed elevated levels of basal alertness reflected in increased metabolic heart rate and body temperature, they also seem to have evolved a highly adapted immune defense against viral infections [3]. Remarkably, bats have increased tolerance to viral infections by exhibiting higher basal levels of innate defence regulators but also a dampened innate immune response upon infection, substitutional to increased responsiveness to viral pathogens [4]. The bat innate immune response appears to be ‘pre-activated’ with higher basal levels of type I interferon expression, in contrast to humans, who are very quick responders to viral infections, but require a lot more dampening of their immune signals afterwards to get back to basal levels. Current research also shows the absence of any AIM2 mediated inflammasome activation [5], dampened NLRP3 mediated inflammasome activation [6] and dampened STING activation [7] in bats upon infection, which are all mediators of a robust type I interferon response. Overall, Wang et al. demonstrated that bats’ response to stress in form of viral infections is more targeted and thus potentially more effective by numerous adaptions and modifications of the innate immune system.

In ‘Viruses and their Hosts’ session, Vinod Sundaramoorthy (CSIRO-Australian Animal Health Laboratories) discussed a novel defence mechanism in neurons against Rabies virus (RABV). RABV is a neurotropic virus, which causes tens of thousands of deaths every year, despite available vaccines [8]. Endemic dog rabies results in an ongoing risk to humans in many resource-limited countries, whereas rabies in wildlife is important in North America and Europe [9]. Requirement for an uninterrupted vaccine cold chain and the high cost of the immunoglobulin component of rabies prophylaxis therapies substantiate the unmet need for novel RABV-specific antivirals [10]. Furthermore, the pathogenesis of RABV relating to viral replication in neurons is not fully understood [11]. Although most of the infection with RABV manifests as the ‘furious’ form, where virus silently spreads through the neuronal axon without any damage, in 20 % of cases it can cause axonal damage in peripheral neurons leading to paralysis [12]. Using a new in vitro microfluidics model for studying synaptically connected neurons, Sudaramoorthy investigated the pathogenesis of different strains of Rabies virus, showing distinct mechanisms at play in determining disease outcomes for each strain. Future efforts to define a key molecular determinant in the viral replication pathway in neurons may provide a novel therapeutic target.

In 2015, an association between Zika virus (ZIKV) infection during pregnancy as a cause of microcephaly and other congenital abnormalities in the developing fetus and newborn, was made [13]. As such, there has been a plethora of recent ZIKV research focused on understanding the pathogenesis of disease, as well as immunity to the virus and the development of vaccines and effective therapeutics. Novel findings pertaining to all of these areas were presented throughout the meeting. Developments in understanding the structure of ZIKV particles was showcased by Shee-Mei Lok (Duke-NUS, Singapore). Lok and colleagues have previously solved a thermally stable 3.7 Å resolution cryo-electron microscopy structure of ZIKV [14], and were able to now show high resolution structures of ZIKV at various stages of viral assembly. Specifically, the organisation of the envelope protein of the virus and changes during assembly and maturation were presented. Previously, Lok’s lab has also published structures of mature and immature dengue virus, providing insight into the viral maturation process [15]. The research from Lok and colleagues will have future implications in designing novel therapeutics and vaccines for ZIKV. Efforts to develop a ZIKV vaccine using virus like particles were presented by Julio Carrera, working with researchers at the Monash University and the University of Melbourne.

In the ‘Pathogenesis and Prevention of Infection’ session, Rosa Coldbeck-Shackley working with Michael Beard at the University of Adelaide, Australia, and also colleagues at the Hudson Institute, presented findings on the importance of interferon-epsilon (IFN-ɛ) in the innate immune response to ZIKV infection. IFN-ɛ is a novel type I IFN, encoded within the type I IFN locus in mice and humans, whose function has recently been characterized [16]. Like other type I IFNs, it acts via IFN-α receptors 1 and 2, activating Interferon stimulated genes (ISGs). However, IFN-ɛ is preferentially expressed by epithelial cells of the female reproductive tract in both mice and humans, and in contrast to viral induced type I IFN expression, IFN-ɛ is hormonally regulated. Other than mosquito-borne transmission, ZIKV is also sexually transmitted [17]. This makes the research presented by Coldbeck-Shackley significant, and concurs with previous studies where IFN-ɛ–deficient mice were more susceptible to infection with sexually transmitted pathogens [16]. Thus, IFN-ε appears to be a potent antipathogen and immunoregulatory cytokine that may be important in combating sexually transmitted infections that represent a major global health and socioeconomic burden.

Also in the ‘Pathogenesis and Prevention of Infection’ session, Allison Abendroth (University of Sydney) presented ‘*Disarming the killer: targeting of natural killer cells by varicella zoster viru*s’. Varicella zoster virus (VZV), is known to infect numerous immune cell types such as T-cells and dendritic cells [18]. Moreover, the virus is able to modulate and manipulate innate and adaptive immune responses to infection to its replicative benefit. VZV infection of dendritic cells dampens type I IFN responses [19] and leads to evasion of CD8 T cell recognition via downregulation of MHC class I expression [20]. In addition, VZV mediated delay in immune responses to infection facilitates establishment of initial primary infection and lifelong latency in neurons.

Most recently, Abendroth and colleagues found that VZV also productively infects natural killer (NK) cells and that NK cells effectively transmit infection to other permissive cell types [21] . The group is now trying to understand, how NK cells, a cell type that normally demonstrates very effective direct and indirect antiviral capacity, is manipulated by VZV infection, leading to impaired cytotoxicity and cytokine responses upon infection and facilitating infection and spread of the virus. Led by the observation that patients with impaired NK cell functionality are highly susceptible to severe and life threatening VZV infection, Abendroth et al. found limited NK cell activation and efficacy upon VZV infection of target cells and characterised differential modulation of ligands normally recognised by activated NK cells [22]. The exact underlying mechanisms, how VZV infection prevents the release of proinflammatory cytokines during infection of NK cells to impair their general function and whether correlations can be drawn to VZV infection of monocytes and macrophages [23], is currently under their investigation, Abendroth stated.

A key highlight at the conclusion of the meeting was a presentation from the Victorian Infection and Immunity Network Young Investigator Prize winner, Simone Park (The University of Melbourne). Park, working alongside Thomas Gebhardt and Laura Mackay, has recently published seminal research investigating skin tissue-resident memory T cells (TRM cells). Specifically, Park has demonstrated how these cells contribute to antiviral immune memory in peripheral tissues, using a Herpes Simplex model of infection [24]. Using an epicutaneous melanoma model, Park and colleagues also demonstrate that TRM cells play protective role in tumor surveillance [25], which has important implications for advancing anticancer immunotherapies.

The organisers are looking forward to celebrating the 10th Lorne Infection and Immunity meeting in 2020 and invite all researchers with an interest in infectious and inflammatory diseases and associated immune responses to participate. For more information: http://www.lorneinfectionimmunity.org/.

## Abbreviations

IFN: Interferon
NK: Natural killer
RABV: Rabies virus
TRM: Cells, tissue-resident memory cells
VZV: Varicella zoster virus
ZIKV: Zika virus
